# Safety and Effectiveness Outcomes between Apixaban Versus Vitamin K Antagonists in Atrial Fibrillation Patients on Dialysis

**DOI:** 10.31083/j.rcm2509321

**Published:** 2024-09-10

**Authors:** Zongwen Guo, Yufan Wang, Xiaoli Ding, Jiying Lai, Yijian Chen

**Affiliations:** ^1^Department of Critical Care Medicine, The First Affiliated Hospital of Gannan Medical University, 341000 Ganzhou, Jiangxi, China; ^2^Queen Mary School, Nanchang University, 330031 Nanchang, Jiangxi, China; ^3^Clinical Laboratory, The First Affiliated Hospital of Gannan Medical University, 341000 Ganzhou, Jiangxi, China; ^4^Department of Hematology, The First Affiliated Hospital of Gannan Medical University, 341000 Ganzhou, Jiangxi, China; ^5^The Endemic Disease (Thalassemia) Clinical Research Center of Jiangxi Province, 341000 Ganzhou, Jiangxi, China

**Keywords:** apixaban, vitamin K antagonists, atrial fibrillation, dialysis, thromboembolism, end-stage kidney disease

## Abstract

**Background:**

Anticoagulant therapy for atrial fibrillation (AF) in patients with end-stage kidney disease (ESKD) undergoing dialysis poses significant challenges. This review aimed to furnish clinicians with the latest clinical outcomes associated with apixaban and vitamin K antagonists (VKAs) in managing AF patients on dialysis.

**Methods:**

Literature from the PubMed and Embase databases up to March 2024 underwent systematic scrutiny for inclusion. The results were narratively summarized.

**Results:**

Six studies were included in this review, comprising the AXADIA-AFNET 8 study, the RENAL-AF trial, and four observational studies. In a French nationwide observational study, patients initiated on apixaban demonstrated a diminished risk of thromboembolic events (hazard ratios [HR]: 0.49; 95% confidence interval [CI]: 0.20–0.78) compared to those on VKAs. A retrospective review with a 2-year follow-up, encompassing patients with AF and ESKD on hemodialysis, evidenced no statistical difference in the risk of symptomatic bleeding and stroke between the apixaban and warfarin groups. Two retrospective studies based on the United States Renal Data System (USRDS) database both indicated no statistical difference between apixaban and VKAs in the risk of thromboembolic events. One study reported that apixaban correlated with a reduced risk of major bleeding relative to warfarin (HR: 0.72, 95% CI: 0.59–0.87), while the other study suggested that apixaban was associated with a decreased risk of mortality compared to warfarin (HR: 0.85, 95% CI: 0.78–0.92). The AXADIA-AFNET 8 study found no differences between apixaban and VKAs in safety or effectiveness outcomes for AF patients on dialysis. The RENAL-AF trial, however, was deemed inadequate for drawing conclusions due to its small sample size.

**Conclusions:**

Currently, the published studies generally support that apixaban exhibits non-inferior safety and effectiveness outcomes compared to VKAs for AF patients on dialysis.

## 1. Introduction

Atrial fibrillation (AF) is a common arrhythmia in patients with end-stage kidney disease (ESKD) [1]. Recent studies have reported a higher incidence of AF in patients undergoing dialysis (hemodialysis or peritoneal dialysis) [2, 3, 4]. AF is associated with a five-fold increased risk of stroke or systemic embolism (SE), and ESKD is also linked to a heightened risk of cardiovascular diseases, including stroke [5, 6]. Therefore, anticoagulant therapy, such as vitamin K antagonists (VKAs) and direct oral anticoagulants (DOACs), is crucial for preventing these complications. However, managing anticoagulation therapy in patients with AF undergoing dialysis presents unique challenges due to altered pharmacokinetics and an elevated risk of bleeding [7, 8].

Among the available anticoagulants, both warfarin and apixaban are widely used and FDA-approved medications suitable for patients with kidney disorders undergoing dialysis [9]. VKAs inhibit the synthesis of vitamin K-dependent clotting factors, thereby indirectly halting the coagulation cascade [10]. However, the emergence of DOACs has resulted in a decline in VKA usage for treating AF patients on dialysis. This transition is attributed to the narrow therapeutic index, frequent monitoring requirements, and potential drug and food interactions associated with VKAs [11]. Observational studies have also indicated that the utilization of conventional VKAs in patients with renal insufficiency may lead to a higher risk of severe bleeding and vascular calcification, potentially contributing to atherosclerosis [12, 13].

Apixaban, a DOAC, selectively targets factor Xa, resulting in a more direct and predictable anticoagulant effect. These mechanistic disparities could potentially impact treatment outcomes, including efficacy and safety, for patients undergoing dialysis. Compared to VKAs, apixaban may offer several advantages due to its rapid pharmacodynamic response, reduced drug interactions, lack of need for consistent clinic monitoring, and ability to be safely administered at fixed doses without pharmacogenetic analysis [14]. Several studies have reported that apixaban is more effective than VKAs in preventing stroke among patients with AF [15, 16, 17]. Concerning bleeding, Reed *et al*. [18] observed a lower incidence of major bleeding in patients treated with apixaban compared to warfarin. Granger and colleagues [14] reported a 7.7% reduction in any bleeding events in the apixaban group compared with that in the warfarin group. Additionally, a comprehensive study demonstrated that apixaban has an equivalent effect to warfarin in preventing thromboembolic events while generally exhibiting a comparable yet improved safety profile in terms of bleeding events [19]. However, despite the bleeding risks associated with medication, individual patient characteristics, such as impaired renal function, can also contribute to increased bleeding. Further investigation is warranted to evaluate the benefit-risk ratio of anticoagulant therapy for AF patients on dialysis, as well as to compare the safety and effectiveness of apixaban with VKAs.

Currently, several studies have specifically investigated the therapeutic efficacy and prognosis of apixaban relative to VKAs in patients with ESKD and AF undergoing dialysis. Therefore, this review aimed to consolidate recent pertinent studies assessing the therapeutic effects of apixaban and VKAs in AF patients with dialysis.

## 2. Methods

### 2.1 Search Strategy

Two authors conducted the literature search independently. The PubMed and Embase databases were systematically searched until March 2024. We sought studies reporting the effectiveness and safety outcomes of apixaban and VKAs in AF patients with ESKD on dialysis. The search primarily employed the following keywords: (1) “atrial fibrillation”, (2) “dialysis” OR “hemodialysis”, (3) “apixaban”, (4) “vitamin K antagonists” OR “warfarin” OR “coumadin” OR “acenocoumarol” OR “phenprocoumon”. Detailed search strategies are provided in **Supplementary Table 1**. No linguistic restrictions were imposed during the literature search. Although this review systematically searched the literature, it is ultimately a narrative review and therefore not registered.

### 2.2 Inclusion and Exclusion Criteria

Studies eligible for inclusion in this review should meet the following criteria: (1) study design: randomized controlled trials (RCTs) or observational cohort studies; (2) population: patients with AF and end-stage renal disease on dialysis (hemodialysis or peritoneal dialysis); (3) groups: apixaban versus VKAs (e.g., warfarin, coumadin, acenocoumarol, phenprocoumon); (4) outcomes: effectiveness and safety outcomes extracted from the original included studies. Publication types, such as reviews, comments, case reports, case series, letters, editorials, and meeting abstracts, were excluded due to insufficient data.

### 2.3 Study Selection

**Fig. 1.**
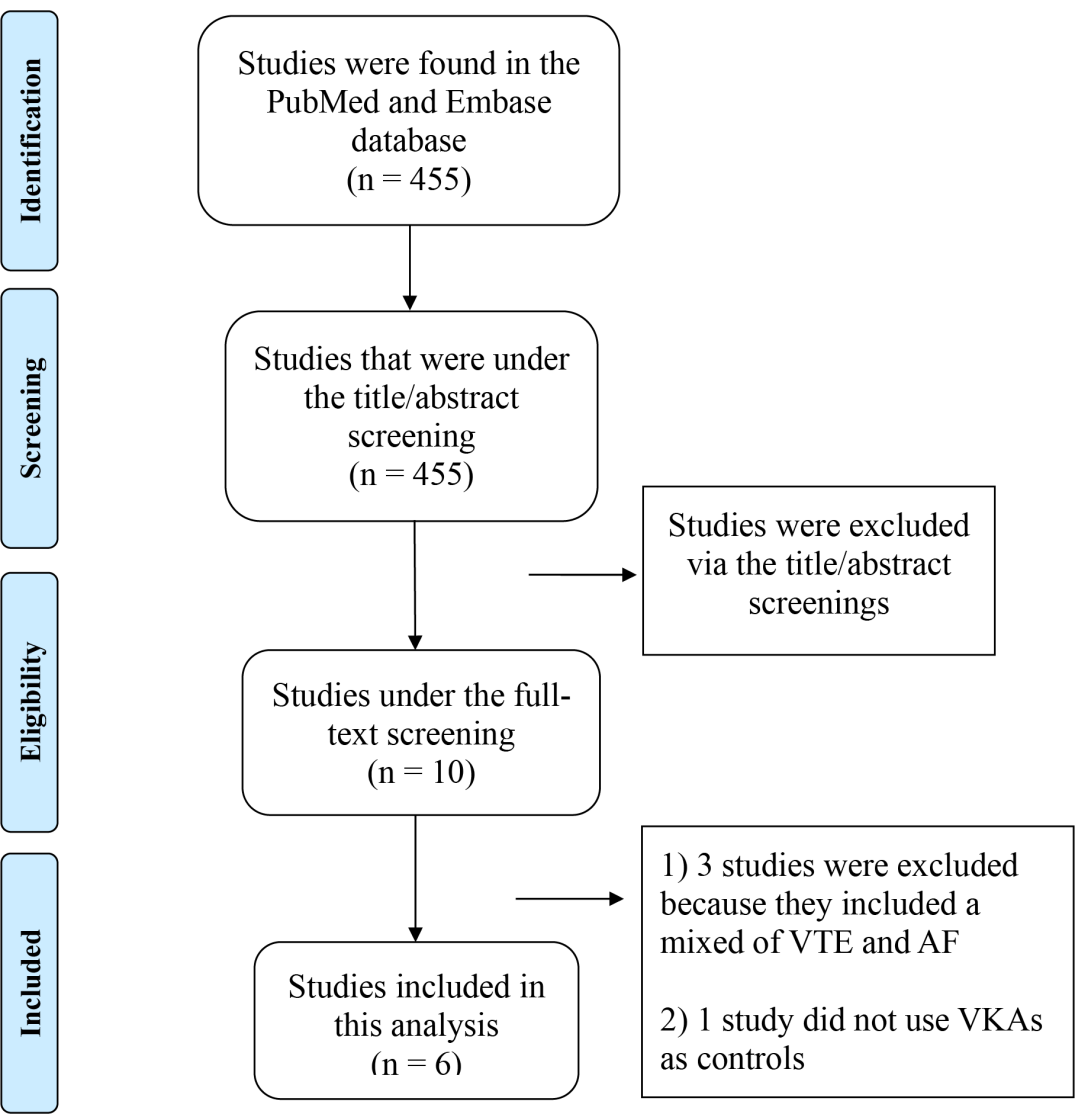
**Flow chart that summarizes the literature search process**. VTE, venous thromboembolism; AF, atrial fibrillation; VKAs, vitamin K antagonists.

The literature search strategies employed in this study are delineated in Fig. 1. Initially, a total of 455 studies were identified in the PubMed and Embase databases. Subsequently, 445 studies were excluded via the title/abstract screenings. After that, 10 studies were under full-text screening, and 4 studies were further excluded because (1) 3 studies included a mix of venous thromboembolism (VTE) and AF [18, 20, 21], and (2) one study did not use VKAs as controls [22]. Consequently, a total of 6 studies (comprising 2 RCTs [23, 24] and 4 observational cohorts [25, 26, 27, 28]) were ultimately included in this review.

## 3. Results

### 3.1 Baseline Characteristics of Included Studies

Table 1 (Ref. [23, 24, 25, 26, 27, 28]) outlines the baseline data from the 6 studies included in this review. It included author names, study design pattern, follow-up duration, sample size, apixaban dosing regimen, baseline characteristics of the study population, and comorbid conditions.

**Table 1.** **Baseline characteristics and demographic information of included studies**.

Refs.	Study design	Apixaban, n	VKAs, n	Dose of Apixaban	Age, years (SD)		Gender, n male (%)		CHA_2_DS_2_VASc score, mean		HTN, n (%)		DM, n (%)		Prior stroke, n (%)		HF, n (%)	
					Api	VKAs	Api	VKAs	Api	VKAs	Api	VKAs	Api	VKAs	Api	VKAs	Api	VKAs
Siontis *et al*. [25]	Observational, US Renal Data System	2351	23,172	Apixaban 5 mg BID (43%), 2.5 mg BID (57%)	68.87 (11.49)	68.15 (11.93)	1280 (54.4)	12,572 (54.3)	5.27	5.24	2342 (99.6)	23,079 (99.6)	1773 (75.4)	17,348 (74.9)	778 (33.1)	7683 (33.2)	7683 (33.2)	778 (33.1)
	Database, 10/2010–12/2015.																	
Wetmore *et al*. [26]	Observational, US Renal Data System	4639	12,517	Apixaban 5 mg BID (51%), 2.5 mg BID (49%)	Overall 66.2 (9.4)		Overall 61.7%		2.5-mg (4.7), 5-mg (4.3)	4.5	2.5-mg (97.2%), 5-mg (96.3%)	95.3%	2.5-mg (79.2%), 5-mg (76.4%)	76.6%	N/A	N/A	2.5-mg (66.1%), 5-mg (63.1%)	65.2%
	Database, 04/2013–12/2018.																	
Moore *et al*. [28]	Observational, 11 acute care hospitals and 5 anticoagulation centers, 2-year follow-up.	53	57	Apixaban 5 mg BID (39%), 2.5 mg BID (61%)	68.74 (10.28)	63.37 (16.18)	29 (54.7)	30 (54.5)	3	3	N/A	N/A	N/A	N/A	17 (32.1)	23 (40.4)	N/A	N/A
Laville *et al*. [27]	Observational, French REIN registry, 01/2012–12/2020.	298	8471	N/A	74 (N/A)	73 (N/A)	64%	63%	N/A	N/A	N/A	N/A	51%	46%	23%	22%	26%	28%
AXADIA-AFNET 8 study [24]	RCT, 462 day follow-up.	48	49	Apixaban 2.5 mg BID	74.7 (8.1)	74.8 (7.9)	31 (64.6)	37 (75.5)	4.50	4.54	N/A	N/A	N/A	N/A	8 (16.7)	8 (16,3)	N/A	N/A
RENAL-AF trial [23]	RCT, 15 month follow-up.	82	72	N/A	69.0 (7.0)	68.0 (7.5)	48 (58.5)	50 (69.4)	4.0	4.0	N/A	N/A	42 (51.2)	47 (65.3)	17 (20.7)	12 (16.7)	43 (52.4)	41 (56.9)

N/A, not applicable; VKA, vitamin K antagonist; RCT, randomized controlled trial; REIN, renal epidemiology and information network; Api, apixaban; HTN, hypertension; DM, diabetes mellitus; HF, heart failure; BID, twice daily.

The retrospective cohort study by Siontis *et al*. [25] included a total of 9404 matched cohorts with ESKD undergoing hemodialysis or peritoneal dialysis. Data were extracted from the United States Renal Data System (USRDS) spanning from October 2010 to December 2015. The study comprised 7053 warfarin users and 2351 apixaban users (44% on 5 mg twice daily (BID), 56% on 2.5 mg BID), with an average age of 68.2 years and 45.7% being female. Another retrospective cohort study by Wetmore *et al*. [26] in 2022 involved 17,156 patients with AF on hemodialysis, also sourced from the USRDS database between April 2013 and December 2018. Among them, 12,517 patients received warfarin treatment, and 4639 patients were prescribed apixaban, with 52% on 5 mg BID and 48% on 2.5 mg BID. The average age of the patients was 66.2 ± 9.4 years, with 38.3% being female.

The French national retrospective study conducted Laville *et al*. [27] utilized data extracted from the French Renal Epidemiology and Information Network (REIN) registry and the French national healthcare system database (SNDS) between January 2012 and December 2020. This study aimed to compare the safety and effectiveness of DOACs versus VKAs for dialysis patients. It included 298 apixaban users and 8471 VKA users for evaluation in its secondary analysis. Finally, the retrospective cohort study by Moore *et al*. [28] analyzed data from electronic health records obtained from 11 acute care hospitals and 5 anticoagulation centers between January 2018 and December 2019, with a 2-year follow-up chart review. In total, 53 patients (32 on 2.5 mg BID and 21 on 5 mg BID) were recruited into the apixaban group, and 57 patients were included in the warfarin group.

The AXADIA-AFNET 8 study, conducted by Reinecke *et al*. [24] spanned from June 2017 to May 2022 and included 97 patients with AF undergoing hemodialysis. The trial randomized 48 subjects to the apixaban group (2.5 mg BID) and the other 47 subjects to the VKA group. The average age of participants was 75 years, with females comprising 30% of the cohort, and the median follow-up time was 462 days. The RENAL-AF trial, led by Pokorney *et al*. [23], was a RCT comparing apixaban versus warfarin in AF patients on hemodialysis. It enrolled a total of 154 patients (82 on apixaban and 72 on warfarin). Among the apixaban patients, 71% received a standard dosing regimen (5 mg BID), while the remaining 29% received reduced dosing (2.5 mg BID) due to age ≥80 years, weight ≤60 kg, or both. The average age of participants was 68 years, with females representing 56% of the cohort. This trial commenced in January 2017 and concluded in January 2019, with an individual therapeutic duration up to 15 months.

### 3.2 Apixaban in AF Patients on Dialysis: Evidence from Retrospective Cohorts

Tables 2,3 (Ref. [23, 24, 25, 26, 27, 28]) delineate the key outcomes from the included observational studies. Siontis *et al*. [25] found that the incidence of stroke or SE was 12.4% in the apixaban group and 11.8% in the warfarin group, with no significant difference in mortality free of stroke or SE between the two groups. The hazard ratio (HR) for treating stroke/SE as a death risk was 0.88 (95% confidence interval [CI]: 0.69–1.12) for apixaban compared to warfarin, indicating no statistically significant difference. Furthermore, the apixaban cohort exhibited a reduced risk of major bleeding compared to the warfarin cohort (HR: 0.72, 95% CI: 0.59–0.87). No significant difference was noted in gastrointestinal (GI) bleeding (HR: 0.86, 95% CI: 0.72–1.02) or intracranial bleeding between apixaban and warfarin. Similarly, there was no significant trend observed in all-cause mortality between the two groups (HR: 0.85, 95% CI: 0.71–1.01). Notably, the standard apixaban group (5 mg BID) exhibited significantly lower risk of stroke or SE (HR: 0.64, 95% CI: 0.42–0.97), major bleeding (HR: 0.71, 95% CI: 0.53–0.95), and mortality (HR: 0.63, 95% CI: 0.46–0.85) compared to the warfarin group. In line with the standard dose group, the reduced apixaban dosage group (2.5 mg BID) exhibited a diminished risk of major bleeding relative to warfarin (HR: 0.71, 95% CI: 0.56–0.91), while there were no notable differences in stroke, SE or mortality. Additionally, there were no statistical variances between the 5 mg BID and 2.5 mg BID apixaban groups regarding the incidence of GI or intracranial bleeding compared to warfarin.

**Table 2.** **Effectiveness outcomes between apixaban and VKAs of included studies**.

Reference	Effectiveness outcomes	Results		HR (95% CI)	*p*-value
		Apixaban	VKAs		
Siontis *et al*. [25]	Stroke or systemic embolism	12.4%/100 PY (n = 81)	11.8%/100 PY (n = 373)	0.88 (0.69, 1.12)	0.29
Wetmore *et al*. [26]	Stroke or systemic embolism	5-mg: 2.0%/100 PY (n = 52)	2.1%/100 PY (n = 424)	5-mg: 0.89 (0.65, 1.21)	N/A
		2.5-mg: 1.9%/100 PY (n = 54)		2.5-mg: 0.85 (0.62, 1.17)	
Moore *et al*. [28]	Hospitalization or emergency department visit for ischemic stroke	7.5% (n = 4)	10.5% (n = 6)	N/A	0.742
Laville *et al*. [27]	Thromboembolism events	9.7% (n = 29)	51.0% (n = 2513)	0.49 (0.30, 0.78)	N/A
AXADIA-AFNET 8 Study [24]	Cardiovascular mortality, myocardial infarction, ischemic stroke, deep vein thrombosis, pulmonary embolism	20.8% (n = 10)	30.6% (n = 15)	N/A	0.508
RENAL-AF trial [23]	Stroke	2% (n = 2)	3% (n = 2)	N/A	N/A
	Death	26% (n = 21)	18% (n = 13)		

PY, patient-years; N/A, not applicable; HR, hazard ratio; CI, confidence interval; VKA, vitamin K antagonist; AXADIA-AFNET, Compare Apixaban and Vitamin K Antagonists in Patients With Atrial Fibrillation and End-Stage Kidney Disease; RENAL-AF, Renal Hemodialysis Patients Allocated Apixaban Versus Warfarin in Atrial Fibrillation. Results from intention-to-treat (ITT) analysis were included.

**Table 3.** **Safety outcomes between apixaban and VKAs of included studies**.

Reference	Safety outcomes	Results		HR (95% CI)	*p*-value
		Apixaban	VKAs		
Siontis *et al*. [25]	Major bleeding	19.7%/100 PY (n = 129)	22.9%/100 PY (n = 715)	0.72 (0.59, 0.87)	< 0.001
	Gastrointestinal bleeding	23.8%/100 PY (n = 155)	23.4%/100 PY (n = 710)	0.86 (0.72, 1.02)	0.09
	Intracranial bleeding	3.1%/100 PY (n = 21)	3.5%/100 PY (n = 111)	0.79 (0.49, 1.26)	0.32
	Death	23.7%/100 PY (n = 159)	24.9%/100 PY (n = 753)	0.85 (0.71, 1.01)	0.06
Wetmore *et al*. [26]	Major bleeding	5-mg: 4.5%/100 PY (n = 127)	6.3%/100 PY (n = 1226)	0.67 (0.55, 0.81)	*p * < 0.05
		2.5-mg: 4.7%/100 PY (n = 117)		0.68 (0.55, 0.84)	*p * < 0.05
	All-cause mortality	5-mg: 24.4%/100 PY (n = 716)	28.8%/100 PY (n = 6096)	0.85 (0.78, 0.92)	*p * < 0.05
		2.5-mg: 28.0%/100 PY (n = 741)		0.97 (0.89, 1.05)	*p * > 0.05
Moore *et al*. [28]	Symptomatic bleeding	39.6% (n = 21)	36.8% (n = 21)	N/A	0.918
Laville *et al*. [27]	Major bleeding	6.0% (n = 18)	14.4% (n = 1220)	0.54 (0.22, 1.32)	N/A
AXADIA-AFNET 8 Study [24]	Major bleeding or all-cause death	48.5% (n = 22)	51.0% (n = 25)	0.93 (0.53, 1.65)	N/A
RENAL-AF trial [23]	Major or clinically relevant nonmajor bleeding	26% (n = 21)	22% (n = 16)	N/A	N/A

PY, patient-years; N/A, not applicable; HR, hazard ratio; CI, confidence interval; VKA, vitamin K antagonist; AXADIA-AFNET, Compare Apixaban and Vitamin K Antagonists in Patients With Atrial Fibrillation and End-Stage Kidney Disease; RENAL-AF, Renal Hemodialysis Patients Allocated Apixaban Versus Warfarin in Atrial Fibrillation. Results from intention-to-treat (ITT) analysis were included.

Another retrospective cohort study by Wetmore and colleagues [26] suggested that there was no significant difference in the incidence of stroke/SE among the label-concordant apixaban group (5 mg BID), the below-label apixaban group (2.5 mg BID), and the warfarin group according to intention-to-treat (ITT) analysis. Both the 5 mg BID apixaban group (HR: 0.67, 95% CI: 0.55–0.81) and the 2.5 mg BID apixaban group (HR: 0.68, 95% CI: 0.55–0.84) demonstrated a decreased risk of major bleeding compared to the warfarin group in the ITT analysis. However, there was no difference in the risk of major bleeding between the 5 mg BID apixaban group and the 2.5 mg BID group (HR: 1.02, 95% CI: 0.78–1.34). Regarding all-cause mortality (ITT analysis), the 5 mg BID apixaban group exhibited a reduced risk compared to the warfarin group (HR: 0.85, 95% CI: 0.79–0.92), whereas no difference was observed between the warfarin group and the 2.5 mg BID apixaban dosing group (HR: 0.97, 95% CI: 0.89–1.05). Similar results were obtained from censored at drug switch or discontinuation (CAS) analysis. Pooled apixaban users did not show any significant difference compared to warfarin in terms of the incidence of stroke/SE. However, apixaban was associated with a lower risk of major bleeding (ITT, HR: 0.69, 95% CI: 0.60–0.80) and decreased mortality compared to warfarin (HR: 0.79, 95% CI: 0.70–0.90) for ITT analysis, although no difference was observed in CAS analysis.

Furthermore, the French observational study [27] reported that 29 patients (9.73%) in the apixaban group and 2513 patients (29.86%) in the VKAs group experienced thromboembolism events (HR: 0.49; 95% CI: 0.20–0.78), indicating a lower risk for effectiveness outcomes with apixaban administration relative to VKAs. However, no statistical difference was observed in major bleeding events between apixaban and VKAs (HR: 0.50; 95% CI: 0.24–1.04). Moreover, in the multi-centered retrospective cohort by Moore *et al*. [28], there was no significant difference between apixaban and warfarin in terms of ischemic stroke events after a 2-year follow-up. Four participants (7.5%) in the apixaban group and six participants (10.5%) in the warfarin group experienced thromboembolic events (*p* = 0.742). Notably, all patients meeting the primary outcomes in the apixaban group underwent 2.5 mg BID. Additionally, there was no disparity in the risk of symptomatic bleeding between the apixaban group and the warfarin groups (apixaban vs. warfarin = 39.6% vs. 36.8%). Thrombocytopenia was experienced by 22.6% of patients in the apixaban group and 21.1% in the warfarin group. Particularly, the discontinuation rate during the two-year follow-up was 73.6% in the apixaban group and 73.7% in the warfarin group, with high mortality contributing to the discontinuation (27 (50.9%) patients in the apixaban group and 25 (43.9%) in the warfarin group died during the study period).

### 3.3 Apixaban in AF Patients on Dialysis: Evidence from RCTs

Tables 2,3 delineate the key outcomes of the included RCTs. In the RCT conducted by Reinecke and colleagues [24], safety outcomes were evaluated based on the occurrence of the first event of major bleeding, clinically associated nonmajor bleeding, or all-cause death, while efficacy outcomes comprised the composite of ischemic stroke, myocardial infarction, deep vein thrombosis, pulmonary embolism, or all-cause death. Regarding the primary composite safety outcome, no significant difference was observed between the apixaban (22 patients, 45.8%) and the VKA group (25 patients, 51.0%), with an HR of 0.93 (95% CI: 0.53–1.65). Among on-treatment events, 18 safety events occurred in the apixaban group and 18 events in the VKA group. Overall, the incidence of the composite primary safety outcome in the full analysis set was 79% in the VKAs treatment group and 59% in the apixaban treatment group. The efficacy outcome events occurred in 10 patients (20.8%) in the apixaban group compared to 15 patients (30.6%) in the VKA group. Furthermore, cumulative incidences of the primary efficacy events in the full analysis set were 54% in the VKA group and 32% in the apixaban treatment group. Additionally, no statistically significant difference was found between apixaban and VKAs in individual events such as major bleeding (10.4% vs. 12.2%), myocardial infarction (4.2% vs. 6.1%), and all-cause death (18.8% vs. 24.5%).

In the study by Pokorney *et al*. [23], the 1-year incidence of major or clinic-related non-major bleeding was 32% in the apixaban group and 26% in the warfarin group (HR: 1.20, 95% CI: 0.63–2.30). Meanwhile, the 1-year incidence of stroke or SE was 3.0% in the apixaban group and 3.3% in the warfarin group. Notably, death emerged as the most prevalent major event in both the apixaban group (21 of 82, 26%) and the warfarin group (13 of 72, 18%). A pharmacokinetic study was also conducted for apixaban on 50 enrolled patients, revealing a median steady-state 12-hour area under the curve of 2475 ng/mL×h (10th to 90th percentiles, 1342–3285) for patients receiving 5 mg dosing and 269 ng/mL×h (10th to 90th percentiles, 615–1946) for those on the 2.5 mg apixaban dosing arm, indicating substantial overlap between the maximum and minimum blood concentrations of apixaban.

## 4. Discussion

Atrial fibrillation stands as the most prevalent clinically managed cardiac arrhythmia globally, inherently linked to an increased risk of thromboembolic events [29]. Factors such as heightened oxidative stress and inflammation represent primary pathogenic contributors to AF, severely impacting atrial structural and electrical remodeling [30]. Chronic obstructive pulmonary disease is also closely correlated with atrial fibrillation due to the increased oxidative stress it causes, which is implicated in compromised diastolic function and abnormal electrocardiogram findings [31]. Notably, epigenetic modifications by microRNAs further exacerbate the persistent occurrence of AF, exacerbating atrial fibrosis and cardiac electrical properties [32]. Additionally, excessive inflammatory stress may also significantly contribute to AF persistence and recurrence [33]. An aberration in calcium handling represents another major pathogenic mechanism, potentially leading to reduced calcium overload in atrial myocytes and subsequent atrial arrhythmic events [34]. Together, these factors play a pivotal role in the initiation and progression of AF, while also facilitating thrombosis formation. Atrial fibrosis and electrical alternations could elicit blood stasis and slow intra-atrial flows, thereby favoring local thrombotic patterns [35]. An inflammatory endothelium, diminished anticoagulant factors, and increased procoagulant factors further contribute to systemic prothrombotic patterns [36]. Therefore, anticoagulant therapy is crucial for patients with AF. However, AF patients undergoing dialysis treatment also face a significantly heightened risk of bleeding [7], presenting a challenge to current anticoagulant therapy for these individuals.

The present understanding of the effectiveness and safety of apixaban and VKAs regarding their application in AF patients on dialysis remains incomplete. Two retrospective cohort studies [25, 26], both utilizing data from the USRDS database across different time periods, compared apixaban to warfarin in this population. Another retrospective study [28] conducted in the United States included patients from multiple centers but with a limited sample size. The French nationwide study [27] drew data from the REIN registry, aiming to assess the anticoagulant efficacy of DOAC therapy and VKAs therapy in dialysis patients. The RENAL-AF trial [23], a pioneering investigation on the effect of apixaban and warfarin for stroke prevention in ESKD patients with AF undergoing hemodialysis, was also conducted in the United States. Additionally, another RCT, the AXADIA-AFNET 8 study [24], assigned patients to either the 2.5 mg BID apixaban group or the VKA phenprocoumon group to evaluate their effectiveness and safety.

Both retrospective studies utilizing USRDS data [25, 26] indicated that apixaban was associated with a decreased risk of major bleeding compared to warfarin in AF patients on dialysis. Standard apixaban dosing (5 mg BID) exhibited advantages in reducing both mortality and thromboembolic events compared to warfarin treatment, while no significant difference in safety and effectiveness outcomes was noted for reduced apixaban dosing (2.5 mg BID) relative to warfarin. Furthermore, findings from the French nationwide registry [27] suggested that apixaban displayed significantly higher efficacy in reducing thrombotic events compared to VKAs, with a non-significant decrease in major bleeding risk. Similarly, the AXADIA-AFNET 8 Study [24] revealed no differences in safety or effectiveness outcomes between apixaban and VKAs in AF patients undergoing hemodialysis. However, the RENAL-AF trial [23] lacked a sufficient sample size to draw adequate conclusions regarding the risk of major or non-major bleeding events comparing apixaban to warfarin. Collectively, these results from the retrospective studies and the RCT studies suggest that there is no evidence to indicate that apixaban therapy is less safe or effective than VKAs.

While only a limited number of studies have delved into investigating the safety and efficacy of apixaban and warfarin in AF patients undergoing dialysis, their pioneering results also highlight various challenges and uncertainties that may exist in future research endeavors. One of the earliest investigations into the effects of apixaban in AF patients receiving hemodialysis was conducted by Pokorney *et al*. [23], a prospective, randomized, open-label, blinded-outcome assessment comparing apixaban to warfarin. Despite providing initial RCT data for apixaban in this population, Pokorney’s study was limited by its small sample size, precluding definitive conclusions. This difficulty may be attributed to concerns voiced by many physicians who deem anticoagulation inappropriate for ESKD patients with AF on hemodialysis, resulting in significant resistance in volunteer recruitment. Consistent with this concern, the RENAL-AF trial by Pokorney reported a notable mortality rate, with 34 patients (22%) deceased, alongside 37 patients experiencing major or nonmajor bleeding events, while 4 patients (3%) suffered from stroke/SE. This high mortality and incidence of bleeding relative to thromboembolism raise questions regarding the optimal strategy for stroke prevention in AF patients on dialysis. A Canadian observational study revealed a hospitalization-related bleeding rate of 7.3% per year among this population, with a slightly higher bleeding rate among patients on anticoagulation (10.9% per year). While the study by Siontis *et al*. [25] suggested a significantly higher major bleeding rate of 22.3% per year among AF patients on hemodialysis receiving anticoagulant treatment. Future investigations are imperative to determine the balance between the benefits of stroke prevention and the risks of major bleeding associated with anticoagulation in influencing all-cause mortality for AF patients on dialysis.

In the context of patient management in atrial fibrillation, the safety profile of non-vitamin K antagonist oral anticoagulants (NOACs) compared to VKAs has been extensively evaluated. NOACs are generally considered superior to VKAs, demonstrating a reduced incidence of intracranial and major bleeding events [37]. A meta-analysis conducted by Silverio *et al*. [38], comprising 22 studies involving 440,281 AF patients, revealed that NOACs were associated with lower risk of intracranial bleeding (HR: 0.46, 95% CI: 0.38–0.58), hemorrhagic stroke (HR: 0.61, 95% CI: 0.48–0.79), and fatal bleeding (HR: 0.46, 95% CI: 0.30–0.72), while exhibiting increased gastrointestinal bleeding (HR: 1.46, 95% CI: 1.30–1.65) relative to VKAs. Consistent with these findings, a network meta-analyses [7] reported that standard-dose DOACs were associated with a significantly reduced hazard of intracranial bleeding (HR: 0.45, 95% CI: 0.37–0.56), but an increased risk of gastrointestinal bleeding (HR: 1.31, 95% CI: 1.08–1.57). Conversely, lower-dose DOACs were linked to a non-significant risk of gastrointestinal bleeding (HR: 0.85, 95% CI: 0.62–1.18) and significantly reduced risk of intracranial bleeding (HR: 0.28, 95% CI: 0.21–0.37) and major bleeding (HR: 0.63, 95% CI: 0.45–0.88), compared to warfarin. This heightened risk of gastrointestinal bleeding associated with DOACs relative to warfarin may be attributed to their incomplete gastrointestinal absorption and dose-dependent variations in anticoagulant intensity at the gastrointestinal mucosa [39, 40]. However, this disadvantage of DOACs in AF patients may be offset by their efficacy in reducing thromboembolic events, intracranial, and fatal bleeding, which are of greater clinical significance. Moreover, the ARISTOPHANES study [41] investigating the safety of oral anticoagulants in nonvalvular AF Patients found that that apixaban demonstrated higher efficacy in reducing stroke/SE (HR: 0.64, 95% CI: 0.58–0.70) and a lower incidence of major bleeding (HR: 0.60, 95% CI: 0.56–0.63) compared to warfarin. Additionally, AF patients with compromised renal function, numerous studies [42, 43, 44] have further indicated a lower risk of major bleeding events with apixaban compared to warfarin, further supporting the, at least non-inferior, clinical safety of apixaban relative to VKAs in this population.

Another RCT, the AXADIA-AFNET 8 study, conducted by Reinecke and colleagues [24] in Germany, yielded no statistically significant differences in both safety and effectiveness outcomes between apixaban and VKAs in patients with AF undergoing hemodialysis. However, despite anticoagulant application, patients with AF on hemodialysis remained at high risk of cardiovascular disease. Several weaknesses are apparent in this study. Firstly, this RCT lacked a third control group with no anticoagulant application, making it challenging to determine the appropriateness of DOACs for AF patients on hemodialysis. Although the high rate of thromboembolic events from experimental results rationalized this bias, the absence of a control group limits the interpretation of the findings. Furthermore, the author noted that the AXADIA-AFNET 8 Study did not enroll an adequate number of patients as originally planned, leading to an inability to conclusively establish the noninferiority of apixaban to VKAs concerning safety in the full analysis set. Additionally, the AXADIA-AFNET 8 Study only employed one regimen of apixaban (2.5 mg BID), potentially restricting the comprehensiveness of the results for comparing apixaban with VKAs regarding their effectiveness and safety. Moreover, a significant proportion of patients with severe or moderate-to-severe conditions declined to participate in this trial, as advised by their family practitioners. This refusal might have introduced a strong bias in population selection and result analysis.

Four retrospective studies [25, 26, 27, 28] demonstrated similar drawbacks in comparing the effectiveness and safety of apixaban and warfarin. A major limitation of their studies is their non-randomized design, inherent in retrospective studies due to the varied conditions under which data is collected from diverse healthcare centers. This lack of randomization introduces inconsistencies and unavoidable confounding factors. Consequently, the results from these retrospective studies should be interpreted as hypotheses rather than providing practical efficacy due to their bias in population selection stemming from their retrospective nature. Another limitation is that these studies only investigated databases from the United States and France, potentially limiting the generalizability of their results to other populations. This drawback may arise from the fact that the current clinical application of DOACs for AF patients on dialysis is predominantly approved in American and European countries. This discrepancy may be influenced by concerns or regulatory processes specific to each jurisdiction, including the need for further clinical evidence or regulatory approval pathways. Therefore, clinicians should be mindful of local regulatory guidelines and consider individual patient factors when prescribing anticoagulation therapy for this specific population. Moreover, two USRDS retrospective studies ascertained the incidence of AF from prior administrative claims [25, 26], potentially leading to some degree of residual misclassification. Additionally, only a small number of patients in their investigations were on peritoneal dialysis, where warfarin might exert better effectiveness than in hemodialysis [45]. This disparity could result in misjudgments in evaluating effectiveness. Furthermore, Laville and colleagues [27] collected data from an administrative database, which hindered them from accessing exact prescriptions of oral anticoagulants for each patient. Moreover, the high discontinuation rate in Moore’s study [28] (73.6% in the apixaban group, 73.7% in the warfarin group) posed challenges in collecting final outcomes and generating data for long-term therapy. Additionally, their small sample size rendered them unable to propose statistically significant results.

Notably, two USRDS retrospective studies suggested no difference in risk of major bleeding between the 2.5 mg and the 5 mg dosages of apixaban [25, 26]. However, contrary to this conclusion, an observational study by Xu *et al*. [46] reported a higher risk of major bleeding in the 5 mg apixaban dosing group (4.9% per year) compared to the 2.5 mg apixaban dosing group (2.9% per year). Additionally, they found no disparity in thromboembolic risk (HR: 1.01, 95% CI: 0.59–1.73) or mortality (HR: 1.03, 95% CI: 0.77–1.38) between 5 mg BID and 2.5 mg BID apixaban dosages. Conversely, Siontis and colleagues [25] demonstrated a significantly lower risk of stroke/SE (HR: 0.61, 95% CI: 0.37–0.98) and death (HR: 0.64, 95% CI: 0.45–0.92) in the 5 mg apixaban group compared to the 2.5 mg dosing group, indicating uncertainty regarding the safety and effectiveness of different apixaban dosages. Pharmacodynamic findings from the RENAL-AF study suggest that plasma concentrations resulting from the 2.5 mg BID dosage assessed in the AXADIA–AFNET 8 study are akin to those seen in individuals without kidney disease, while concentrations resulting from the 5 mg BID dosage are comparable to those observed in patients with chronic kidney disease. This implies that an individual’s renal excretion function could significantly influence the pharmacodynamic outcomes of apixaban. Moore *et al*. [28] also noted that some patients were maintained on the 2.5-mg BID dosage solely due to severe renal dysfunction, rather than meeting two out of three criteria for dose adjustment [28]. Additionally, the 5 mg BID dosage demonstrated no correlation with a higher risk of bleeding. Furthermore, the current apixaban dosing recommendation remains divergent between the European Medicines Agency and the US Food and Drug Administration. The European Medicines Agency proposes that patients suffering from severe renal impairment (estimated creatinine clearance between 15 to 29 mL/min) should receive reduced apixaban dosing, while the FDA indicates a different pattern concerning weight, age, and serum creatinine concentration [47]. These results suggest that further investigation into the dosage regimen of apixaban is necessary to minimize the risk of increased bleeding to the maximum extent possible.

Collectively, compared to VKAs, apixaban demonstrates non-inferiority in both safety and effectiveness for preventing ischemic stroke/embolic events in AF patients undergoing dialysis. In view of the potentially favorable risk-benefit ratio for patients having dialysis, apixaban may serve as a promising alternative regimen to VKAs. Urgent large-scale, multi-centered RCT studies are necessary to thoroughly investigate the effectiveness and safety of apixaban and warfarin in this specific population, especially as more countries approve the clinical use of DOACs, including apixaban. Specifically, the impact of anticoagulants on stroke prevention and the risk of major bleeding warrants reevaluation. Furthermore, the new guidance of the recommended apixaban dosage pattern needs to be reenacted. Future investigations should include a larger proportion of patients with severe or moderate-to-severe conditions to achieve a more comprehensive understanding of the outcomes.

## 5. Limitations

This narrative review has several limitations. Firstly, the conclusion supporting apixaban as a non-inferior therapeutic option to VKAs for AF patients on dialysis is subject to several drawbacks. The limited number of studies investigating the use of apixaban and VKAs in this particular population constrain the robustness of our conclusion, which relies on a narrative assessment of six studies. The inconsistency of information generated by individual studies with different research types and experimental criteria precludes quantitative analysis across studies. Importantly, the absence of statistical analysis with pooled data can introduce potential bias. Secondly, with four studies conducted in the USA and the remaining two in Europe, the generalizability of our recommendation may not extend to a global population. Another significant limitation arises from the reluctance of most general practitioners to prescribe apixaban or warfarin for patients with severe or moderate-to-severe kidney disorders, potentially biasing our conclusions. Moreover, one retrospective study [28] and two RCTs [23, 24] yielded no statistically significant results, suggesting no superiority of apixaban over VKAs in terms of safety and effectiveness. Furthermore, two RCTs included in this review recruited a relatively inadequate patient cohort into their studies, which made their experimental results unpersuasive.

## 6. Conclusions

Current published studies generally suggest that apixaban has non-inferior safety and effectiveness outcomes compared to VKAs in AF patients on dialysis. The potentially favorable risk-benefit profile of apixaban implies its potential as a preferred alternative to VKAs for thrombotic event prevention. Urgent attention is required for large and multi-centered RCTs to delve deeper into the safety and effectiveness outcomes of apixaban versus warfarin in this patient population.

## Availability of Data and Materials

Data sharing is not applicable as no new data were generated or analyzed in this study.

## Supplementary Material

Supplementary material associated with this article can be found, in the online version, at https://doi.org/10.31083/j.rcm2509321.
