# Supplementary material for: Safety and Effectiveness Outcomes between Apixaban Versus Vitamin K Antagonists in Atrial Fibrillation Patients on Dialysis
Source: Rev Cardiovasc Med. 2024 Sep 10;25(9):321. doi: 10.31083/j.rcm2509321 (PMC11440421; doi:10.31083/j.rcm2509321)
Supplement: Supplementary file 1 [file 2153-8174-25-9-321-s1.zip › RCM23161-Supplementary Material-V3.pdf]

Supplementary Table 1. The search strategies of our current meta-analysis until March 10, 2024

Database	Keywords	No.
**PubMed**		
#1	'atrial fibrillation'	112,016
#2	‘dialysis’ OR ‘hemodialysis’	238,756
#3	'apixaban'	5,790
#4	‘vitamin K antagonist’ OR ‘warfarin’ OR ‘coumadin’ OR ‘acenocoumarol’ OR ‘phenprocoumon’	44,886
#5	#1 and #2 and #3 and #4	93
**Embase**		
#1	'atrial fibrillation'	243,244
#2	‘dialysis’ OR ‘hemodialysis’	373,330
#3	'apixaban'	22,841
#4	‘vitamin K antagonist’ OR ‘warfarin’ OR ‘coumadin’ OR ‘acenocoumarol’ OR ‘phenprocoumon’	126,828
#5	#1 and #2 and #3 and #4	362
